# Study of dose distribution in a human body in international space station compartments with the tissue-equivalent spherical phantom

**DOI:** 10.1093/jrr/rrt220

**Published:** 2014-03

**Authors:** Vyacheslav A. Shurshakov, Raisa V. Tolochek, Ivan S. Kartsev, Vladislav M. Petrov, Igor V. Nikolaev, Svetlana I. Moskalyova, Vladimir I. Lyagushin

**Affiliations:** 1 State Research Center of Russian Federation Institute of Biomedical Problems, Russian Academy of Sciences; 2 Scientific Center “SNIIP”, Moscow, Russia; 3 Rocket Space Corporation “Energia”, Moscow region, Russia

**Keywords:** space radiation, spherical phantom, passive and active detectors, effective dose

## Abstract

Space radiation is known to be key hazard of manned space mission. To estimate accurately radiation health risk detailed study of dose distribution inside human body by means of human phantom is conducted. In the space experiment MATROSHKA-R, the tissue-equivalent spherical phantom (32 kg mass, 35 cm diameter and 10 cm central spherical cave) made in Russia has been used on board the ISS for more than 8 years. Owing to the specially chosen phantom shape and size, the chord length distributions of the detector locations are attributed to self-shielding properties of the critical organs in a real human body. If compared with the anthropomorphic phantom Rando used inside and outside the ISS, the spherical phantom has lower mass, smaller size and requires less crew time for the detector installation/retrieval; its tissue-equivalent properties are closer to the standard human body tissue than the Rando-phantom material. Originally the spherical phantom was installed in the star board crew cabin of the ISS Service Module, then in the Piers-1, MIM-2 and MIM-1 modules of the ISS Russian segment, and finally in JAXA Kibo module. Total duration of the detector exposure is more than 1700 days in 8 sessions.

In the first phase of the experiment with the spherical phantom, the dose measurements were realized with only passive detectors (thermoluminescent and solid-state track detectors). The detectors are placed inside the phantom along the axes of 20 containers and on the phantom outer surface in 32 pockets of the phantom jacket. After each session the passive detectors are returned to the ground. The results obtained show the dose difference on the phantom surface as much as a factor of 2, the highest dose being observed close to the outer wall of the compartment, and the lowest dose being in the opposite location along the phantom diameter. Maximum dose rate measured in the phantom is obviously due to the galactic cosmic ray (GCR) and Earth' radiation belt contribution on the ISS trajectory. Minimum dose rate is caused mainly by the strongly penetrating GCR particles and is observed behind more than 5 g/cm^2^ tissue shielding. Critical organ doses, mean-tissue and effective doses of a crew member in the ISS compartments are also estimated with the spherical phantom data. The estimated effective dose rate is found to be from 10 to 15% lower than the averaged dose on the phantom surface as dependent on the attitude of the critical organs.

The spherical phantom proved its effectiveness to measure the critical organ doses together with the effective dose in-flight and if supplied with active dosimeters can be recommended for future exploratory manned missions to monitor continuously the effective dose.

